# Diagnosing vestibular hypofunction: an update

**DOI:** 10.1007/s00415-020-10139-4

**Published:** 2020-08-07

**Authors:** Dmitrii Starkov, Michael Strupp, Maksim Pleshkov, Herman Kingma, Raymond van de Berg

**Affiliations:** 1 Division of Balance Disorders, Department of Otorhinolaryngology and Head and Neck Surgery, Maastricht University Medical Centre, Maastricht, The Netherlands; 2 Faculty of Physics, Tomsk State Research University, Tomsk, Russia; 3 German Center for Vertigo and Balance Disorders, Ludwig Maximilians University, Munich, Germany; 4 Department of Neurology, Ludwig Maximilians University, Munich, Germany; 5 Maastricht University ENT Department, P. Debyelaan 25, 6229 HX Maastricht, The Netherlands

**Keywords:** Bilateral vestibulopathy, Unilateral vestibulopathy, Video-oculography, Video-head impulse test, Caloric testing, Vestibular evoked myogenic potentials, Dynamic visual acuity, Perceptual threshold

## Abstract

Unilateral or bilateral vestibular hypofunction presents most commonly with symptoms of dizziness or postural imbalance and affects a large population. However, it is often missed because no quantitative testing of vestibular function is performed, or misdiagnosed due to a lack of standardization of vestibular testing. Therefore, this article reviews the current status of the most frequently used vestibular tests for canal and otolith function. This information can also be used to reach a consensus about the systematic diagnosis of vestibular hypofunction.

## Introduction

Vestibular hypofunction (also vestibulopathy, vestibular dysfunction, -hyporeflexia, -loss, -failure, -deficiency), i.e. a unilateral or a bilateral vestibulopathy, is a heterogeneous disorder of the peripheral and/or rarely central vestibular system leading typically to disabling symptoms such as dizziness, imbalance, and/or oscillopsia [1–3]. It affects up to 95 million adults in Europe and the USA [4]. Unfortunately, vestibular hypofunction is often missed or misdiagnosed. Fortunately, nowadays vestibular function of the semicircular canals and the otolith organs can be quantified, but there are still some diagnostic challenges [5–10]. For instance, no consensus has been reached regarding standardization of vestibular testing yet. This leads to a large variability in normative and pathologic cut-off values. Furthermore, not many vestibular laboratories have obtained their own normative values [11]. For the purpose of standardization, this article reviews the current status of the most clinically used vestibular tests. The topo-diagnostic value of posturography, despite its broad application, is already a long time under dispute, and for that reason not addressed in this review [12]. Finally, a new method for self-motion perception will be discussed.

## Video-oculography and electro-oculography

Nowadays, video-oculography (VOG) is used as a routine method in clinical practice to quantitatively measure eye movements, whereas EOG has rarely applied anymore [13].

Using an infrared video camera, VOG detects eye movements by analyzing 2D images of the eye that is illuminated by infrared LEDs. The position of the pupil is calculated and used to track the horizontal and vertical eye movements, generally up to sample frequencies of 250 Hz or less [15]. Resolution and accuracy of VOG vary with image quality but are generally < 1° in 2D over a gaze range of a minimum of ± 25° horizontal and ± 20° vertical, as long as the pupil is fully visible for the camera. Detection of torsional eye movements is based on the detection of rotation of the iris structure around the pupil center. This technique often fails as the image of the iris varies with gaze due to its 3D structure. Another possible alternative is to use a contact lens with markers [16]. However, this method is not common in practice and its clinical value is still to be determined.

Electro-oculography (EOG) is based on detecting the corneo-retinal potential [14–16] using electrodes placed around the eyes. An EOG resolution in 2D of typically < 1° can be obtained over a gaze range of ± 30° horizontal and ± 20° vertical (although linearity only holds up to an eccentric gaze of 15–20°). The most frequently used sample frequency is 50–250 Hz. Accuracy (detection of absolute eye position) is limited due to substantial drift that often occurs. Frequent recalibrations are required as corneo-retinal potentials and EOG amplitudes vary by a factor of 4–5 with low versus bright ambient light intensity. Above 100 Hz the signal-to-noise-ratio often decreases by an increasing contribution of other electrophysiological signals such as EMG [17].

VOG and EOG systems both meet requirements for clinical use in terms of their accuracy and sensitivity of approximately 1°. There does not seem to be a “best method”, but VOG can be easily applied in clinical practice and is, therefore, most often used. The preferred method of choice depends on how clinicians and/or technicians weigh the pros and cons of each technique with respect to their own requirements and patient population.

## The video-head impulse test (vHIT)

The video-head impulse test (vHIT) is able to quantitatively assess the vestibulo-ocular reflex (VOR) of all six semicircular canals in the high-frequency domain and it can be used in acute, episodic and chronic vestibular syndromes [6, 18, 19]. However, it does not purely test one vestibular organ: some contribution from the other side remains [6]. vHIT is a much more sensitive and specific test than the clinical HIT [20]. The commercially available vHIT-devices differ regarding patient comfort, the accuracy of pupil detection, and methods for quantifying the VOR.

The accuracy of pupil detection can be affected by incorrect camera adjustment, poor calibration, blinking, eyelashes, narrow eyelids, poor illumination, mascara, spontaneous nystagmus, and goggle slippage. These factors can cause recording artifacts that might not be detected by the software and which could negatively influence the reliability of test outcomes [21–25]. Training of examiners is, therefore, imperative, since it significantly reduces artifacts [26]. The number of impulses required to achieve reliable results can be reduced to two in the case of artifact-free traces, which is especially important when testing very young children with relatively little attention [27]. The VOR is quantified by calculating gain. VOR gain is the measure that illustrates to which extent eye movements (produced by the VOR) compensate for head movements. However, all commercially available devices use different gain calculation methods, which can lead to significant discrepancies in results [28, 29]. Two methods have been proposed to standardize gain calculation and to lower variability in results [30], but they are not (yet) routinely implemented in the clinic. Gain is also influenced by head velocity (higher velocity, lower gain) [31], target distance (shorter distance, higher gain) [32]), and (in the case of goggles) a direction bias for the side on which the camera is placed (higher gains for the side with the camera [25]). It is, therefore, advised to standardize these variables as much as possible in the clinic. If no normative data is available, absolute gain values below 0.8 can be considered pathological [33].

The vHIT is able to detect corrective saccades appearing during and after head impulses (covert and overt saccades, respectively). An earlier timing [34, 35] and a higher level of grouping (clustering regarding timing, quantified using the PR-score) of saccades might be indicative of compensation and might be correlated with a lower handicap in patients with vestibular hypofunction [36–40]. However, corrective saccades do not only reflect clinically relevant vestibular hypofunction or compensatory mechanisms: small saccades also appear in healthy subjects, especially with increasing age [41].

The Suppression Head Impulse Paradigm (SHIMP) was proposed to overcome the problem of covert saccades regarding gain calculation [42, 43]. SHIMP differs from vHIT with respect to the target: it moves along with the head. In this case, the presence of corrective saccades indicates the presence of vestibular function [42], while the absence of corrective saccades indicates impaired vestibular function [44]. VOR gain with SHIMP is significantly lower than with vHIT, which might (partially) be explained by age and VOR inhibition strategies during SHIMP [45, 46]. SHIMPs significantly reduce covert saccades, but not all of them. Furthermore, vHIT alone seems to be sufficient for detecting bilateral vestibulopathy regardless of the presence of covert saccades (van Dooren et al., in preparation).

## Testing of Dynamic Visual Acuity

The visual acuity during dynamic conditions (e.g. walking) is called “dynamic visual acuity” (DVA). An impaired VOR (especially bilaterally) causes blurred vision during head movements. This can result in loss of DVA.

The DVA loss is quantified by the difference in visual acuity in static and dynamic conditions, which is measured using optotype charts or computerized DVA systems [7, 47]. The dynamic conditions can involve walking or active/passive head movements while sitting or standing. In case of impaired VOR, DVA loss is generally higher for passive head movements than for active head movements [48, 49]. Contradictory evidence exists regarding the influence of age on DVA: it is either weak or absent [50–53]. Nevertheless, age decreases the ability to accomplish DVA testing on a treadmill in bilateral vestibulopathy patients and healthy subjects [53].

Recently, a new test for high-frequency DVA was proposed: the functional head impulse test (fHIT) [54–56]. During fHIT, the test subject is placed in front of a computer screen and head impulses are applied in the tested plane. When head acceleration exceeds a predefined threshold, a Landolt ring optotype appears for a predefined short time on the screen. After the impulse, the subject is instructed to choose its orientation using a keyboard. The percentage of correct answers is used as the output of the test. The test has shown effectiveness when evaluating acute unilateral vestibulopathy [57] and the effect of a prototype vestibular implant [58]. Although fHIT moderately correlates with oscillopsia severity, no correlation was observed between fHIT and the DVA test on a treadmill [59]. These tests seem complementary and do not substitute for each other.

DVA is a functional outcome of all systems involved: VOR, the oculomotor system, and central processing of signals [59]. For instance, internal feed-forward commands can mediate gaze [60], gait stabilization strategies can help to reduce head oscillations [61], and covert saccades improve DVA in patients with unilateral vestibulopathy [34, 62]. Therefore, DVA testing is mainly suited for evaluating the functional state of the vestibular system and compensation strategies, not for diagnosing peripheral vestibular deficits.

## Caloric testing

Caloric testing is a widely used method to selectively assess vestibular function on each side in the low-frequency domain (~ 0.003 Hz), using bithermal (30 °C and 44 °C) caloric irrigations with water (the preferred stimulus) or air [5, 8, 63]. To optimize stimulation, horizontal canals are aligned with the vertical plane by asking the test subject in a supine position to tilt the head 20°–30° [64]. Irrigations of sufficient volume (> 250 ml) should last at least 30 s and can be performed in any order [65–67]. For the sake of standardization, it is advised to use cold irrigation first on the right, followed by cold on the left, then warm on the right, and finally warm on the left. A 5 min interval between the four successive irrigations can be used to avoid residual effects of the previous irrigation [68]. The slow phase velocity (SPV) of the caloric nystagmus is measured for each irrigation. In symptomatic patients, the sum of the bithermal maximum peak SPV < 6°/s can be considered a diagnostic criterion for bilateral vestibulopathy and the sum of bithermal maximum peak SPV on each side between 6 and 25°/s for presbyvestibulopathy (when also age ≥ 60 years) [1]. The upper limits for both vestibular asymmetry and directional preponderance can be set at 20% when no normative data is available [65, 69]. Poor attention, poor alertness, visual suppression, and unreliable eye movement detection often lead to false-positive findings of vestibular hypofunction [5].

Complete vestibular areflexia cannot be identified using ice water calorics, since the test mostly only evaluates low-frequency horizontal canal function [70–72]. Moreover, ice water calorics itself might induce an irrelevant latent spontaneous nystagmus in a non-specific way [5].

## Rotatory chair testing

Rotatory chair tests are generally used to assess horizontal semicircular canal function in the low- and middle-frequency domains. Two types of tests are mainly performed: the Torsion Swing Test (TST) and the Velocity Step Test (VST).

The TST is divided into the Sinusoidal Harmonic Acceleration Test (SHAT), which involves a single frequency sinusoidal stimulus, and the Pseudo-Random Rotation Test (PRRT), which involves sinusoidal stimuli with different frequencies. VST uses a slow acceleration (e.g. ≤ 2°/s^2^) to reach a constant velocity (often 100°/s) followed by an abrupt deceleration (e.g. 200°/s^2^). The VST mainly tests the excited canal, though a contribution of the contralateral canal to the total response still remains [73]. The VST is believed to be closer to the frequency spectrum of most natural head movements than TST [5, 9, 74]. Eyes should be open during testing since eye closure reduces the VOR response [75].

The outputs for SHAT are gain (ratio of slow phase eye velocity to chair velocity), phase (time relation between eye and chair velocities), and directional preponderance (asymmetry in magnitude/gain for left and right rotations), whilst for VST the time constant (time for nystagmus to decay to 37% of its peak magnitude) and gain are most relevant [9]. Since the gain is frequency-dependent, each frequency tested by SHAT or PRRT has its own normative values [76–78]. Phase and time constant are very stable parameters: no large inter-laboratory differences are observed [79]. A reduced gain and/or time constant reflect a unilateral or bilateral vestibulopathy, attention deficit, or visual suppression. A high gain and/or long time constant indicate hypersensitivity (anxiety and or hyperventilation during the test, or central pathology leading to disinhibition) [80, 81]. In addition, the product of gain and time constant seems to better reflect the impairment of the vestibular system than gain and time constant alone [82]. Abnormalities of phase and time constant can point to peripheral (e.g. bilateral vestibulopathy) and/or central vestibular disorders. The presence of a directional preponderance indicates a dynamic VOR asymmetry. This is often seen in uncompensated peripheral vestibular disorders and central vestibular disorders and provides insights into the central processing of vestibular input from both labyrinths [5, 9, 74, 77, 83–89].

## Vestibular evoked myogenic potentials

Vestibular evoked myogenic potentials (VEMP) are believed to reflect the otolith function [10, 90].

Air-conducted sound or bone-conducted vibration of the skull induces otolith vestibular responses, resulting in VEMP, which can be recorded using electromyography. Two types of VEMP are currently measured: cervical VEMP (cVEMP) and ocular VEMP (oVEMP). cVEMP mainly evaluate saccular function by measuring the inhibitory response from the ipsilateral sternocleidomastoid muscle. Therefore, the muscle should be contracted during the test. However, differences in muscle contraction can lead to inter- and intrasubject variabilities in response, hindering thorough evaluation [91–94]. Multiple methods have been proposed that effectively reduce variability, although none of them are (yet) widely applied in clinical practice [93, 94]. oVEMP mainly evaluates utricular function by measuring the excitatory response from the contralateral inferior oblique extra-ocular muscle. Standardized upward gaze is necessary to bring the eye muscles in close contact with the electrodes placed below the eyes [10, 90]. Regarding stimuli, air-conducted sound (cVEMP) and bone-conducted vibration (oVEMP) are the preferred stimuli to detect vestibular hypofunction, although air-conducted sound is preferred to detect vestibular hyperfunction (e.g., superior semicircular canal dehiscence syndrome). For air-conduction, obtained results should be corrected for the present air–bone gap in cases with ipsilateral conductive hearing loss [95]. The air-conducted and bone-conducted stimuli mostly involve 500 Hz stimuli presented at a rate of 5 Hz to obtain optimal responses, although VEMP can be tested at different frequencies to obtain more insights into specific disease patterns [96] (e.g., testing a range from 250 to 1000 Hz). Furthermore, increasing the stimulation rate from 5 to 13 Hz has been shown to produce reliable cVEMP thresholds, while decreasing testing time and subject discomfort [94].

Electromyographic responses of VEMP include two peaks of vestibular origin, which appear at approximately 13 and 23 ms in cVEMP, and at approximately 10 and 15 ms in oVEMP. Peaks appearing later in time have mixed and/or different origins including vestibular, stretch reflex and cochlear [97, 98]. Outcome parameters used for VEMP are the presence of the response, the threshold (in dB), peak-to-peak amplitude (µV), peak latency (ms), and interaural asymmetry ratio. The testing paradigm and interpretation of VEMP are not yet standardized [99–102]. For correct interpretation, it is strongly advised to obtain age-matched normative data [11], since with age the amplitudes and response rates decline [103]. This implies that absent responses also appear in non-symptomatic healthy individuals, especially above the age of 60 years old [104]. The role of VEMP in clinical practice has been investigated extensively regarding diagnostics, prognosis, and monitoring of vestibular disorders [105, 106].

The most important clinical application of the VEMP is the syndromes of the third mobile window, including superior canal dehiscence syndrome (SCDS, see below) [106–108]. The relevance of VEMP in Menière’s disease, unilateral and bilateral vestibulopathy, vestibular migraine, BPPV, and auditory neuropathy is very limited or not relevant [104, 109–116]. In SCDS, VEMP amplitudes are increased and VEMP thresholds are lowered on the affected side(s), as a result of a third mobile window [117]. To diagnose SCDS, using oVEMP amplitudes higher than 16.7 µV as cut-off point results in a sensitivity of 100% and a specificity of 89% [118]. Regarding cVEMP thresholds, 2000 Hz tone burst stimuli show the best diagnostic accuracy, with sensitivities equal to or higher than 92% and a specificity of 100% [119]. Taking into account all the existing evidence about the use of VEMP to diagnose SCDS, oVEMP seems to be more sensitive and specific than cVEMP [108].

## Perceptual threshold testing

Self-motion perception was first measured by Mach in the 19th century [120]. Currently used methods to measure self-motion perceptual thresholds involve devices such as hydraulic or electric moving platforms [121, 122] or sleds [123]. To test self-motion perceptual thresholds, the subject is seated on a chair mounted on the platform or sled. Visual, auditory and somatosensory input are decreased as much as possible by, e.g., testing in darkness, wearing headphones, and covering skin surfaces [124]. The platform or sled then accelerates into the tested plane of motion, with the desired stimulus parameters (magnitude, frequency, etc.). After each stimulus, the subject has to indicate whether the movement was perceived. The main outcome parameter is the self-motion perceptual threshold representing the minimal value of a physical stimulus that can still be perceived [125, 126]. There are two types of perceptual thresholds: detection thresholds (motion is perceived: yes/no), and recognition thresholds (type and direction of motion).

In healthy subjects, self-motion perceptual thresholds are higher than horizontal VOR-thresholds, indicating a higher sensitivity of the brainstem than vestibulothalamic pathways [127]. Furthermore, self-motion perceptual thresholds increase after the age of 40 [121, 122], are frequency-dependent (lower thresholds at higher frequencies) [124], decrease with visual input [128], depend on stimulus profile [129, 130] and some thresholds might be affected by vestibular disorders such as Menière’s disease (higher thresholds) and vestibular migraine (lower thresholds) [131–133]. The peripheral vestibular system strongly contributes to self-motion perceptual thresholds (especially rotations), as shown by significantly higher thresholds in patients with bilateral vestibulopathy compared to a control group [133–135]. One of the disadvantages of testing self-motion perception is the substantial time needed to complete testing for one subject (several hours). Recently, a faster method to determine self-motion perceptual thresholds was proposed, which facilitates testing of 12 motion types within 1 h. The clinical value of tests for vestibular perception is not yet fully determined. However, since they can be of direct functional relevance, they might develop in the future into the “speech audiogram” for vestibular disorders [5, 121].

## Detecting vestibular hypofunction: a proposal

To reliably detect vestibular hypofunction, normative laboratory values should be obtained for each test (if possible) and technicians should be trained [11].

When vestibular hypofunction is suspected, it might be recommended to start with vHIT due to its low burden for the test subject. If vHIT results are abnormal, no other vestibular testing is necessary. However, in case of normal vHIT results, performing caloric testing might be advisable, since caloric testing seems to be more sensitive than vHIT in detecting vestibular hypofunction in some vestibular disorders, in particular Menière’s disease [136–139]. Furthermore, a dissociation between caloric testing and vHIT might be present, especially in cases with endolymphatic hydrops due to altered mechanics of the inner ear [140–143]. In case of bilateral vestibulopathy, rotatory chair testing can be added to increase the specificity of testing (not sensitivity) [80, 144] and to help to determine residual vestibular function [82], since the responses to rotatory chair testing are often better preserved than the responses to vHIT or caloric stimulation [144]. Dynamic visual acuity testing is recommended for evaluation of the functional state of the vestibular system as well as compensatory processes occurring over time. VEMP is currently only advised for detecting superior canal dehiscence syndrome.

## Conclusion

Most recently published literature involves refinement or development of vestibular laboratory tests. However, no worldwide consensus has been reached yet on standardized testing procedures and normative values for any of the tests discussed in this article. Standardization will most likely improve the reliability and reproducibility of vestibular laboratory test results.

## Data Availability

Not applicable Not applicable.
